# Understanding the role of SGLT2 inhibitors in glycogen storage disease type Ib: the experience of one UK centre

**DOI:** 10.1186/s13023-022-02345-2

**Published:** 2022-05-12

**Authors:** Rebecca K. Halligan, R. Neil Dalton, Charles Turner, Katherine A. Lewis, Helen R. Mundy

**Affiliations:** 1 Inherited Metabolic Diseases, Evelina London Children’s Hospital, London, SE1 7EH UK; 2 WellChild Laboratory, Evelina London Children’s Hospital, London, UK

**Keywords:** Glycogen storage disease type Ib, Empagliflozin, SGLT2 inhibitors, 1,5-Anhydroglucitol, Neutropaenia, Neutrophil dysfunction

## Abstract

**Background:**

Glycogen storage disease type Ib (GSD Ib) is a severe disorder of carbohydrate metabolism due to bi-allelic variants in *SLC37A4*. It is associated with neutropaenia and neutrophil dysfunction, which has recently been attributed to the accumulation of 1,5-anhydroglucitol-6-phosphate (1,5AG6P) within neutrophils. Treatment with sodium-glucose co-transporter-2 (SGLT2) inhibitors, such as empagliflozin, is a novel therapy that reduces 1,5-anhydroglucitol (1,5AG) in plasma.

**Results:**

We report our experience in treating 8 paediatric GSD Ib patients with empagliflozin with a cumulative treatment time greater than 12 years. Treatment with a median dose of 5 mg (0.22 mg/kg height weight) of empagliflozin resulted in improvement in bowel health, growth, and laboratory parameters. Plasma 1,5AG levels reduced by a median of 78%. Baseline 1,5AG levels in our cohort were higher than in adult patients with GSD Ib. Hypoglycaemia on empagliflozin treatment occurred in 50% of our cohort.

**Conclusion:**

We report the largest single centre cohort of GSD Ib patients treated with empagliflozin to date. Treatment with SGLT2 inhibitors is a novel and favourable treatment option for neutropaenia and neutrophil dysfunction in GSD Ib. We suggest a low starting dose of empagliflozin with careful titration due to the risk of hypoglycaemia. The interpretation of 1,5AG levels and their role in treatment monitoring is yet to be established, and requires ongoing research.

## Background

Glycogen storage disease type Ib (GSD Ib, MIM#232220) is a severe disorder of carbohydrate metabolism affecting the final step in the pathways of both glycogenolysis and gluconeogenesis [1]. The *SLC37A4* gene encodes the glucose-6-phosphate transporter (G6PT), which is responsible for the transportation of glucose-6-phosphate from the cytoplasm to the endoplasmic reticulum (ER) where it is oxidized to glucose [2]. GSD Ib is characterized by fasting hypoglycaemia, hepatomegaly, poor growth, anaemia, renal disease and an increased risk of hepatic adenomas [3, 4]. Biochemical findings typically include hyperlactatemia, hyperuricaemia, hyperlipidaemia and iron deficiency.

GSD Ib patients have neutropaenia and neutrophil dysfunction causing recurrent infections, periodontal inflammation and inflammatory bowel disease (IBD) [5]. The neutropaenia and neutrophil dysfunction in GSD Ib persists throughout life, and is not ameliorated by liver transplantation [6–9]. This is likely to impact on quality of life (QoL) with studies finding that patients with GSD Ib typically reported a lower QoL than patients with GSD Ia [10, 11]. An international priority setting partnership with the James Lind Alliance has listed the treatment of neutropaenia and infections, and management of IBD in GSD Ib as a top research priority [12].

Recently, the neutropaenia and neutrophil dysfunction seen in GSD Ib has been attributed to a build-up of 1,5-anhydroglucitol-6-phosphate (1,5AG6P) within neutrophils [13]. Plasma 1,5-anhydroglucitol (1,5AG) is an analog of glucose, and enters cells where it is phosphorylated to 1,5AG6P and is then transported into the ER by G6PT and dephosphorylated. Accumulation of 1,5AG6P within the cytoplasm of the neutrophils inhibits hexokinases that are required for the first step of glycolysis, thereby impairing the activity of the neutrophil and leading to apoptosis [13, 14].

Renal sodium-glucose co-transporter-2 (SGLT2) inhibitors, such as empagliflozin, are approved treatments in type 2 diabetes mellitus, and function by increasing glucose excretion in urine. SGLT2 is also responsible for the reabsorption of 1,5AG. SGLT2 inhibitors were shown to decrease plasma 1,5AG and restore a normal neutrophil count in a murine model with a phenotype similar to the neutrophil impairment in GSD Ib [14]. Published reports from the few GSD Ib patients treated with empagliflozin have been positive, with improvements in neutrophil counts, wound healing and bowel health [15–17]. Treatment with granulocyte colony-stimulating factor (GCSF) for neutropaenia has been reduced or discontinued in many patients. Importantly, no major side effects have been reported to date [15–17].

We report the experience of one UK centre in managing 8 GSD Ib patients with empagliflozin, and provide commentary on possibilities and pitfalls.

## Methods

### Leukocyte function assessment

Plasma for neutrophil and lymphocyte function testing was collected at the commencement of treatment in 4 patients, and was repeated at least 1 month later. Neutrophil oxidative burst from patient and control cells was assessed by dihydrorhodamine (DHR) flow cytometry following stimulation with phorbol myristate acetate (PMA), N-formyl-methionyl-leucyl-phenylalanine (fMLP) and *Escherichia Coli* (*E. Coli*) [18]. Lymphocyte subsets and a functional assay following stimulation with phytohemagglutinin (PHA) were measured in patients and controls [19]. Measurement of a full blood count was performed every second week following treatment commencement for the first 2 months, and then monthly thereafter.

### Biochemical and haematological testing

All 8 patients were electively admitted to hospital for monitoring upon commencement of therapy. Twice daily urinalysis was performed on treatment commencement and with any change in dose to ensure glucosuria. Patients were electively admitted periodically throughout the year for biochemical profiling, in accordance with the practice of our centre. Venous glucose and lactate levels were performed prior to every meal and snack during inpatient admissions. Assessment of a lipid profile, urate, iron studies and liver function tests were taken prior to starting therapy and periodically thereafter as part of routine monitoring in GSD Ib.

### 1,5-Anhydroglucitol assessment

Plasma for 1,5AG measurement was collected prior to treatment commencement and at regular intervals while on treatment. This was measured using stable isotope dilution on tandem mass spectrometry (MSMS). Urine for 1,5AG measurement was collected on several patients, which enabled calculation of the fractional excretion of 1,5AG. Anonymized plasma 1,5AG levels from adult GSD Ib patients were compared to levels in our paediatric cohort.

### Clinical assessment

Growth was regularly assessed, along with a review of symptoms related to oral health, skin disease, infections and bowel health. Paediatric Ulcerative Colitis Activity Index (PUCAI) scoring was measured prior to treatment commencement and at regular intervals on treatment in 6 patients. PUCAI scoring was chosen by virtue of its noninvasiveness, ease of use and high rate of correlation with professional endoscopic scoring systems [20].

## Results

### Demographics and growth

Three boys and five girls with a median current age of 7.79 years (range 1.5 to 15.83 years) were commenced on empagliflozin with a cumulative treatment time of 12.32 years. The clinical and biochemical data of our 8 patients are presented in Table 1. Treatment with empagliflozin resulted in an improvement in growth parameters, with the median standard deviation for height, weight and body mass index (BMI) all moving closer to the mean using UK-WHO growth charts.

**Table 1:** Clinical and biochemical data at baseline and on treatment

Patient ID	1		2		3		4		5		6		7		8		Median	
Current age (years)	7.0		15.17		8.58		4.33		15.42		15.83		1.5		6.75		7.79	
Time on treatment (years)	1.67		1.25		1.83		2.33		0.75		1.83		0.58		2.08		1.75	
Daily dose (mg/kg htwt)	0.18		0.09		1.28		0.38		0.24		0.09		0.21		0.24		0.22	
Empagliflozin treatment	Pre	Post	Pre	Post	Pre	Post	Pre	Post	Pre	Post	Pre	Post	Pre	Post	Pre	Post	Pre	Post
Weight (Z-score)	− 0.54	− 0.09	2.04	1.9	0.93	0.9	0.84	0	− 1.4	− 0.76	0.1	0.05	1.1	1.34	− 0.34	− 0.45	0.47	0.03
Height (Z-score)	− 0.75	− 0.07	0.43	0.4	− 1.33	− 1.9	− 1.3	− 1	− 3.9	− 3.9	− 0.96	− 0.39	− 1.2	− 0.92	− 0.96	− 1.08	− 1.08	− 0.96
BMI (Z-score)	− 0.1	− 0.11	2.1	1.97	2	2.2	2.3	0.68	0.95	1.56	0.64	0.43	N/A	N/A	0.39	0.23	0.95	0.68
ANC (× 10^9^/L)	2	1	0.1	0.6	1.4	0.7	0.2	0.6	N/A	1.7	0.1	0.6	0.4	0.4	0.4	0.5	0.4	0.6
Haemoglobin (g/L)	107	126	90	123	101	116	105	130	N/A	144	110	140	96	113	103	126	103	126
Iron (μmol/L)	15.3	14.7	3.1	10.7	3.7	8.7	3.1	10.5	N/A	11	8.6	14.5	8.6	6.2	4.7	8.4	4.7	10.6
Urate (μmol/L)	0.47	0.16	0.33	0.25	0.41	0.38	0.39	0.25	N/A	0.16	0.35	0.25	0.35	0.26	0.27	0.14	0.35	0.25
Triglycerides (mmol/L)	6.26	2.81	0.96	0.8	2.72	6.3	2	5	N/A	2.53	1.37	1.49	3.16	2.35	1.92	2.6	2	2.57
PUCAI score	10	10	35	10	55	40	30	0	N/A	N/A	20	5	N/A	N/A	5	10	25	10
Daily GCSF (μg/kg htwt)	1.75	0	N/A	N/A	3.46	0	1.77	0	N/A	N/A	N/A	N/A	N/A	N/A	N/A	N/A	1.77	0

ANC: absolute neutrophil count; GCSF: granulocyte colony-stimulating factor; PUCAI: Pediatric Ulcerative Colitis Activity Index—Severe > 65, moderate 35–64, mild 10–34, in remission < 10. Haemoglobin—normal range < 110 g/L under 6 years, < 115 g/L > 6. Iron—normal range 14-25 μmol/L). Urate—normal range 120–430 μmol/L. Triglycerides – normal range 0.6–1.9 mmol/L

### Empagliflozin dosing and side effects

All patients were commenced on a once daily dose of empagliflozin given in the evening, which increased to twice daily dosing after several weeks in all patients. The current doses are depicted in Table 1 and are presented as milligrams per kilogram of height weight (mg/kg htwt), which is defined as the proportionate body weight in kilograms for the same height percentile. Our centre prefers to calculate carbohydrate intake and drug doses in height weight for our GSD patients as we feel this is more accurate given that there is typically a degree of adiposity. Patients received a median dose of 5 mg (0.22 mg/kg htwt) given in two divided doses, which is a proportionately lower dose than that given in other centres (0.3–0.7 mg/kg/day) [14, 15, 17]. Four patients (50%) experienced hypoglycaemia upon commencement of empagliflozin requiring treatment. One patient had significant symptomatic hypoglycaemic events including an overnight hypoglycaemic seizure. Another patient had loss of previously established metabolic control, as defined by elevated urate, triglycerides and pre-feed lactate levels. These patients were managed by lowering the dose of empagliflozin and adjusting diet therapy.

Additional reported side effects included 1 episode of balanitis and 2 episodes of urinary tract infection. One patient described excessive thirst.

### Leukocyte function assessment

Neutrophil oxidative burst measured by DHR flow cytometry in 3 of the 4 patients was abnormal at baseline compared to controls. All 4 patients had clinical evidence of neutrophil dysfunction with multiple bacterial infections, low iron status and an elevated PUCAI score. Repeat of the neutrophil oxidative burst assay was normal compared to controls in all 4 patients on treatment. Lymphocyte subsets were abnormal in 1 patient at baseline with an increase in double negative and gamma delta T cells. All 4 patients had clinical evidence of lymphocyte dysfunction with frequent viral upper respiratory tract infections. Repeat lymphocyte function testing on all 4 patients was unchanged on treatment.

### Biochemistry and haematology

Baseline biochemical data and data on treatment are presented in Table 1. All patients were anaemic prior to starting empagliflozin, with a median haemoglobin level of 103 g/L. This resolved in all patients after starting treatment, with a median haemoglobin level at last measurement for each patient of 126 g/L. Prior to treatment, 7 out of 8 patients were on treatment for iron deficiency with a median iron level of 4.7 μmol/L. The median iron level on treatment was 10.6 μmol/L and all patients were able to cease iron supplementation.

The absolute neutrophil count (ANC) increased from a median of 0.4 × 10^9^/L to 0.6 × 10^9^/L. Three patients were on treatment with granulocyte colony-stimulating factor (GCSF), with a median dose of 1.77 μg/kg htwt/day (range 1.75–3.46 μg/kg htwt/day), which was later ceased in all patients. Clinically, patients and their families reported a decrease in infections and an improvement in oral health, typically within the first 2 weeks of treatment commencement.

Urate levels improved from a median of 0.35 μmol/L to a median of 0.25 μmol/L. Two patients were on treatment with allopurinol prior to empagliflozin, with one able to cease and one able to decrease their dose. Median triglyceride levels remained stable on treatment.

### 1,5-Anhydroglucitol

**Fig. 1:**
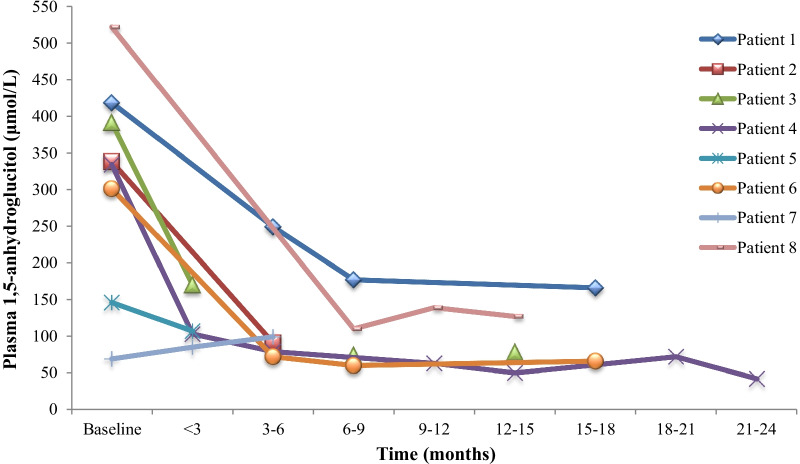
Plasma 1,5-anhydroglucitol levels over time

Plasma 1,5AG levels are presented in Fig. 1, with a median reduction of 78% on treatment with empagliflozin. Due to the Covid-19 pandemic, patient monitoring was performed infrequently; however, a rapid reduction of > 55% was seen in 2 patients within 2–3 weeks after commencement. Plasma levels of 1,5AG in our youngest patient were unrecordable at diagnosis, low at baseline (68.5 μmol/L), and increased slightly on treatment (99 μmol/L).

**Fig. 2:**
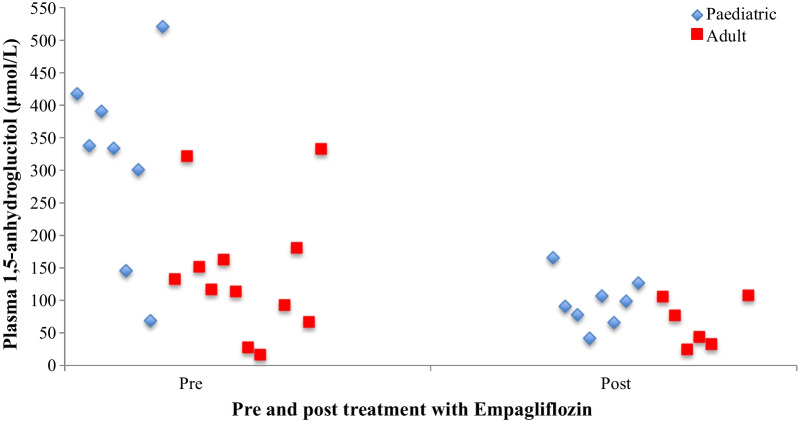
Plasma 1,5-anhydroglucitol levels over time in paediatric and adult patients

Urine 1,5AG levels enabled calculation of the fractional excretion (FE) of 1,5AG. Pre treatment FE of 1,5AG in 4 patients was less than 0.09%. Post treatment FE of 1,5AG was greater than 1% in most patients (range 0.3–3.35%). Measurement of plasma 1,5AG in a number of anonymized adult patients with GSD Ib was lower at baseline (median 125 μmol/L) in comparison to our 8 paediatric patients (median 336 μmol/L), as shown in Fig. 2.

### Clinical assessment

A stable or improved PUCAI score were reported in all patients except one, with a score of 5 prior to treatment commencement rising to a score of 10. Although not formally quantified, a dramatic clinical improvement was seen in all patients within the first few weeks of starting empagliflozin: optimized gum health with decreased mouth ulceration and gingival bleeding, decreased upper respiratory tract infections and a decrease in the number of loose stools. One child went from having multiple loose stools per day to requiring regular treatment for constipation.

## Discussion

Subcutaneous GCSF has traditionally been the mainstay of treatment for neutropaenia and neutrophil dysfunction in GSD Ib; however, this may cause splenomegaly along with a possible increased risk of acute myeloid leukaemia or myelodysplastic syndrome [5, 21]. Treatment with SGLT2 inhibitors, such as empagliflozin, are therefore a promising alternative which has the added benefit of oral administration, avoiding painful subcutaneous injections. Monitoring of our cohort’s ANC revealed only a modest increase in the median ANC which remained below the normal range, consistent with some previous case reports [14]. However, improvement clinically and in neutrophil function tests enabled us to wean and ultimately cease GCSF in all patients. Formation of a gastrostomy for enteral feeding has been contraindicated in GSD Ib patients due to poor wound healing; however, two of our patients have now successfully undergone this procedure [6]. We performed sophisticated immune response studies on 4 of our patients; however, these are expensive and not readily available and we do not feel that they add much to the clinical evaluation.

The benefits of empagliflozin in our cohort extend beyond improvement in neutrophil function. All 8 patients had been anaemic prior to treatment and most were on iron supplementation. Anaemia resolved in all patients and they were able to cease iron supplementation. We hypothesize that this is due to enhanced bowel health secondary to improved neutrophil function leading to an improvement in iron absorption. We monitored PUCAI scores in 6 of our patients, with an improvement seen in 4/6 patients.

Short stature is a common feature in GSD Ib and pleasingly in our cohort, we observed the median Z-score for height increase towards the population mean, which corresponded with a decrease in the median Z-score for weight towards the population mean and an improvement in median Z-score for BMI. The median urate levels also improved, which resulted in one patient ceasing allopurinol. This may be related to a decrease in serum 1,5AG levels, with previous studies finding that serum uric acid levels are closely related to serum 1,5AG levels, which is independent of glucosuria [22]. Alternatively, this may be reflective of improved metabolic control. Median triglyceride levels remained stable on treatment. Long-term follow-up and more research are required in this area to accurately interpret these findings.

SGLT2 inhibitors in patients with GSD Ib have been used off-label and reported in only a few patients. Hypoglycaemia is a known side effect of SGLT2 inhibitors, along with an increased risk of urinary tract infections and balanitis secondary to glucosuria [14]. Wortmann et al. reported an episode of mild hypoglycaemia in 1 patient shortly after commencing empagliflozin, with no symptomatic hypoglycaemic episodes seen in the other reported patients [14–17]. Alarmingly, 50% of our cohort experienced hypoglycaemic episodes following commencement on empagliflozin, with one patient having hypoglycaemic seizures. These were managed by decreasing the dose of empagliflozin by 20–30%, and by individually adjusting dietary therapy to provide a slightly increased carbohydrate intake following a dose of empagliflozin. We postulate that this may be because our centre maintains very tight dietary control for our GSD patients, with an aim to try and keep carbohydrate intake as low as possible. Protocols for the commencement of empagliflozin in patients with GSD Ib have been shared internationally; however, we have used lower doses than those that are suggested. Unpublished data from this author has found evidence of multiple morning hypoglycaemic episodes in a patient with GSD Ib post liver transplant following treatment with empagliflozin, which resolved with the implementation of a milk drink prior to bedtime (R Halligan, unpublished observations). We favoured twice daily dosing in our cohort due to inconsistent glucosuria seen over a 24-h period, and to try and minimise the risk of hypoglycaemia from high doses.

Serum 1,5AG has been used as a marker of acute and short term glycaemic control in type 2 diabetes mellitus for some time [23]. 1,5AG will typically increase from a depleted state with improved glycaemic control, and reference ranges have been reported in small groups of children with and without type 1 diabetes mellitus [24]. However, use of SGLT2 inhibitors in diabetic patients obviates the premise of 1,5AG measurement due to decreasing serum 1,5AG levels by inhibiting reabsorption of 1,5AG in renal tubules. Many labs are now assessing 1,5AG in patients with GSD Ib on treatment with SGLT2 inhibitors. Combined measurement of 1,5AG in plasma and urine enables calculation of the fractional excretion of 1,5AG, with modest decreases (< 5%) in the fractional reabsorption of 1,5AG resulting in drastic decreases in plasma steady-state levels [25].

In our cohort, the median baseline 1,5AG level was 336 μmol/L, which reduced to a median level of 95 μmol/L on treatment. Lower baseline levels were seen in our youngest, untreated patient, and in our laboratory adult cohort, in whom compliance with dietary therapy may be substandard. We hypothesize that plasma 1,5AG may be a marker of energy-dependent proximal tubulopathy, and that in untreated patients or in those who are metabolically unstable, the baseline 1,5AG levels will be lower, even though these individuals continue to experience neutropaenia and clinical evidence of neutrophil dysfunction. This is supported by the well-described proximal renal tubular dysfunction in sub-optimally treated individuals with GSD Ib [2, 26]. Therefore, the establishment of reference ranges for plasma 1,5AG in GSD Ib patients is difficult, and the role of plasma 1,5AG in disease monitoring remains unclear. We do however believe that the calculation of fractional excretion of 1,5AG may be a useful marker of treatment response to empagliflozin.

## Conclusion

We report the largest single centre cohort to date of GSD Ib patients treated with empagliflozin, with a cumulative treatment time greater than 12 years. SGLT2 inhibitors are a favourable treatment option for neutropaenia and neutrophil dysfunction in GSD Ib, and result in improved bowel and gum health with an overall improvement in laboratory markers. However, there is a risk of hypoglycaemia with SGLT2 inhibitors and we therefore recommend a low dose at commencement with careful titration to optimal dosing. The interpretation of 1,5AG levels and their role in treatment monitoring is yet to be established, and requires ongoing research.

## Data Availability

Not applicable.

## Abbreviations

SGLT2: Sodium-glucose co-transporter 2
GSD Ib: Glycogen storage disease type Ib
1,5AG6P: 1,5-Anhydroglucitol-6-phosphate
1,5AG: 1,5-Anhydroglucitol
mg/kg htwt: Milligrams per kilogram of height weight
GCSF: Granulocyte colony-stimulating factor
IBD: Inflammatory bowel disease
ANC: Absolute neutrophil count
PUCAI: Paediatric ulcerative colitis activity index
